# Agricultural trade policies and child nutrition in low- and middle-income countries: a cross-national analysis

**DOI:** 10.1186/s12992-019-0463-0

**Published:** 2019-03-15

**Authors:** Kafui Adjaye-Gbewonyo, Sebastian Vollmer, Mauricio Avendano, Kenneth Harttgen

**Affiliations:** 1 Innovative Methods & Metrics for Agriculture and Nutrition Actions, Tufts University Friedman School of Nutrition Science and Policy, 150 Harrison Avenue, Boston, MA 02111 USA; 2 Institute of Advanced Studies, University College London, Gower Street, London, WC1E 6BT UK; 3 Harvard T. H. Chan School of Public Health, 677 Huntington Avenue, Boston, MA 02115 USA; 4 University of Göttingen; Center for Modern Indian Studies, Waldweg 26, Altbau 1.118, 37073 Göttingen, Germany; 5 Department of Global Health and Social Medicine, King’s College London, Strand Campus, Strand, London, WC2R2LS UK; 6 ETH Zürich, NADEL Center for Development and Cooperation, Clausiusstrasse 37, 8092 Zurich, Switzerland

**Keywords:** Nutrition status, Trade, Agriculture, Policy, Food prices, Nominal rate of assistance, Liberalization

## Abstract

**Background:**

There has been growing interest in understanding the role of agricultural trade policies in diet and nutrition. This cross-country study examines associations between government policies on agricultural trade prices and child nutrition outcomes, particularly undernutrition.

**Methods:**

This study links panel data on government distortions to agricultural incentives to data from 212,258 children aged 6 to 35 months participating in Demographic and Health Surveys from 22 countries between 1991 and 2010. Country fixed-effects regression models were used to examine the association between within-country changes in nominal rates of assistance to tradable agriculture (government price distortions as a percentage of original prices) and child nutritional outcomes (height-for-age, weight-for-age, and weight-for-height Z-scores) while controlling for a range of time-varying country covariates.

**Results:**

Five-year average nominal rates of assistance to tradable agriculture ranged from − 72.0 to 45.5% with a mean of − 5.0% and standard deviation of 18.9 percentage points. A 10-percentage point increase in five-year average rates of assistance to tradable agriculture was associated with improved height-for-age (0.02, 95% CI,0.00–0.05) and weight-for-age (0.05, 95% CI: 0.02–0.09) Z-scores. Improvements in nutritional status were greatest among children who had at least one parent earning wages in agriculture, and effects decreased as a country’s proportion of tradable agriculture increased, particularly for weight-for-age Z-scores.

**Conclusions:**

Government assistance to tradable agriculture, such as through reduced taxation, was associated with small but significant improvements in child nutritional status, especially for children with a parent earning wages in agriculture when the share of tradable agriculture was not high.

**Electronic supplementary material:**

The online version of this article (10.1186/s12992-019-0463-0) contains supplementary material, which is available to authorized users.

## Background

Child undernutrition has severe health and social consequences, impacting child mortality, economic productivity, cognitive development, and risk of chronic disease in adulthood [1–5]. Reducing undernutrition has therefore been a long-standing goal of international development policy [3, 6, 7]. Yet, despite many efforts to reduce undernutrition over the past few decades, progress has been less than optimal. As of 2016, nearly one-quarter of the world’s children under age five were stunted [7]. Childhood undernutrition remains the leading risk factor for the burden of disease in Sub-Saharan Africa [8], and Asia is home to the majority of stunted children [7].

Given the suboptimal improvements in nutritional status globally, increasing attention is being paid not only to nutrition-specific interventions, but also to interventions in other sectors, such as agriculture [9–13]. Sustainable Development Goal 2, for example, has renewed the commitment to reduce undernutrition and has also tied this to sustainable agriculture [7]. Agricultural producers, systems, and outputs can all impact undernutrition and other health outcomes through taxes, subsides and other policy interventions. These can affect the incomes and labor of agricultural producers and in turn, household members’ energy expenditure, time use, and ability to afford nutritious food and other health-promoting amenities. They can also have consequences for nutrition through their impacts on the quantity, quality, diversity, price, and distribution of food available for consumers [12, 14].

Agricultural policy, like policies in other sectors, has undergone a process of trade liberalization—policies aimed at increasing free trade by opening markets and reducing trade restrictions—since the mid-1980s. Trade liberalization has been promoted and adopted globally as a means of economic growth and development and a central component of economic globalization [15–17]. Structural adjustment programs, the 1994 General Agreement on Tariffs and Trade, and the formation of the World Trade Organization (WTO) in 1995 furthered the cause of trade liberalization. However, these policies, particularly as they relate to agriculture, have also been accused of enhancing social and economic injustices and having potentially negative effects on poverty in low- and middle-income countries (LMICs) [15, 18–25]. The empirical research on trade liberalization’s effects on poverty suggest that, while liberalization may lead to reductions in poverty on average this may only be in the long run, and the poor may not always be able to protect themselves from negative consequences or capitalize on the benefits [15, 18, 21, 26–29]. Furthermore, not all countries may see gains from liberalization [30]. Concerns about global inequities in trade and liberalization policies will likely remain, especially given the recent failure of negotiations for the World Trade Organization’s Doha Round which had aimed to address some of these issues, particularly in relation to agriculture [24, 31, 32].

### Trade liberalization, nutrition and health

There are multiple pathways through which agricultural trade policy may affect health and nutrition. Liberalization of agricultural policies can impact food prices, supply and availability, thus directly affecting nutritional status by changing the types and amount of food purchased and consumed. Alternatively, food price changes can affect revenues for agricultural producers, thereby indirectly impacting health and nutrition through household incomes and poverty status [28, 29, 33, 34]. Effects may therefore differ for agricultural consumers and producers [19, 20, 26, 35, 36]. Because agriculture is still the primary employer of the world’s poor, and because of low demand elasticities for food, limited transmission of international to local prices, and limited price changes, income pathways may play a larger role in undernutrition, particularly in economically-disadvantaged groups [12, 28]. Much of the agriculture and nutrition work to date has focused less on these income pathways than on the supply and consumption side, however [12, 13, 28, 37].

### Methodological challenges

Isolating the effects of trade policies from those of other simultaneous policies and secular trends is difficult [34, 38]. Some of the early research on trade policies in relation to nutrition consisted of country case studies of specific liberalization programs or food price changes, such as structural adjustment programs (SAPs) [29, 36, 39, 40]. These studies often used time before and after the implementation of SAPs as an indicator of the policies themselves. However, unobserved factors may be responsible for the time patterns observed in these studies. Also, SAPs typically included multiple components, such as reduced government spending, and were often accompanied by development and food aid that may independently affect health. Moreover, the implementation of SAPs and trade liberalization policies in many countries was often precipitated by other factors that may affect health, such as economic crises or low economic growth [29, 38].

Recent work has also turned its attention toward the effects of liberalization of unhealthy products on overnutrition (overweight and obesity) in addition to undernutrition [41–47]. These studies often use trade volumes or tariff rates as measures of trade liberalization. Yet, trade volumes are also influenced by factors other than trade policy, including geography. Furthermore, tariff rate data fail to account for non-tariff barriers that influence trade [48, 49], and they typically only apply to imports to a country, thus ignoring policies directed at exports, which have historically been used in many African countries [50].

When it comes to agricultural trade however, the World Bank’s Updated National and Global Estimates of Distortions to Agricultural Incentives, 1955 to 2011 [51], offer a potential measurement solution in a metric known as the nominal rate of assistance to agriculture (NRA), which may help to evaluate the effects of changes in agricultural trade policy on undernutrition. The NRA is defined as “the percentage by which government policies have raised gross returns to farmers above what they would be without government intervention” [50]. It is expressed as the ratio of the price distortion on the agricultural product (due to policies such as tariffs, taxes, subsidies, etc.) to the undistorted price of the product. This metric incorporates border market measures (tariffs/taxes or subsidies on imports and exports); the effects of quantitative trade restrictions, such as import bans, as well as dual or multiple foreign exchange rate systems; domestic price distorting-measures such as production subsidies or production taxes; as well as government distortions to prices of farm inputs such as fertilizer. NRA values near zero correspond to liberalized policies, while positive values indicate assistance to domestic agriculture (e.g., tariffs on imported agricultural goods or subsidies on local crops), and negative values represent taxes on local agriculture (e.g., export taxes) [52]. NRAs can be calculated and summed for various agricultural products, such as tradable and non-tradable goods, and can also be compared to NRAs for non-agricultural products to measure relative rates of assistance.

In many LMICs, NRAs have typically been negative, reflecting taxation of or bias against local agriculture. This has largely been driven by fairly high taxation of exportables while import-competing crops have generally received some support in the form of subsidies, et cetera. However, since the 1980s, LMICs have seen sizeable declines in taxation of exportables, while positive support of importables has also declined slightly. This has resulted in NRAs generally becoming less negative overall [50, 53].

One study which has used these data to examine pathways from agricultural policy to nutrition is an analysis by Webb and Block [54]. They combined Distortions to Agricultural Incentives data with data from Demographic and Health Surveys and other sources to examine associations between relative rates of assistance to agriculture, rural population share, income per capita, and national nutrition rates. They found government support to agriculture to be associated with increased income and reduced poverty, predictors of nutrition status. The study—like many others on the effects of trade, agricultural policies, and liberalization on poverty, nutrition, and health—did not examine potential differential effects of these policies among different population subgroups. Examining how such policies may affect various segments of the population differentially may help to elucidate the different mechanisms through which the policies act and may shed light on disparities.

### Objectives

This study aims to address some of the aforementioned research gaps by combining agricultural distortions data with demographic and health microdata to assess the association between agricultural policy changes and child nutritional outcomes (height-for-age, weight-for-age, and weight-for-height). The cross-country analysis focuses specifically on NRAs to agricultural products that are considered tradable, namely exports and import-competing products [52, 55]. We employ a fixed-effects design, reducing the influence of time-invariant confounding factors, and use microdata to examine potential interactions between the policy measures and children’s parental occupation (e.g., non-agricultural, self-employed in agriculture, wage-earning agriculture). Such interactions of effect by parental occupation may point to the relative importance of income versus consumption pathways linking agricultural trade policies to nutrition. We hypothesized that increases in NRAs to tradable agriculture would lead to larger improvements in nutritional status among children in agricultural households compared to those from non-agricultural households.

## Methods

### Data sources

Country-level data on agricultural policy measures were drawn from the World Bank’s Updated National and Global Estimates of Distortions to Agricultural Incentives, 1955 to 2011 [51]. Country-level demographic, economic, and governance indicators were derived from the World Development Indicators dataset, the KOF Index of Globalization, and the Polity IV dataset.

Individual-level data on child undernutrition were obtained from the Demographic and Health Surveys (DHS). The DHS are nationally-representative surveys conducted in LMICs and focusing on maternal and child health, nutrition, demographics, HIV, and other health indicators [56].

### Sample

**Fig. 1:**
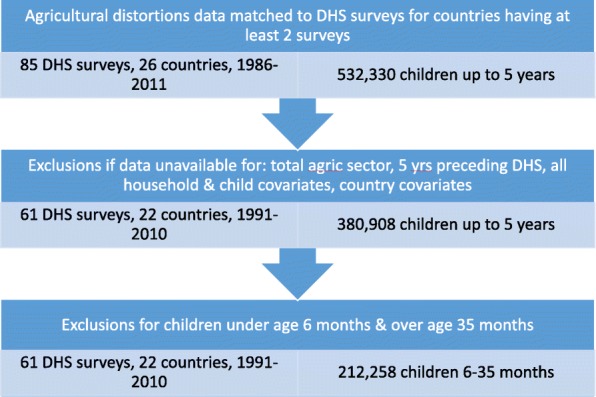
Flowchart of sample selection

Data from the Updated National and Global Estimates of Distortions to Agricultural Incentives, 1955 to 2011 were linked to 85 DHS from 26 countries between 1986 and 2011 for which there were at least two DHS with agricultural distortions data corresponding to the year of the DHS. Surveys were then excluded from the analysis if: a) agricultural distortions estimates were not available for the total agricultural sector or for the five years preceding the DHS sample; b) they did not collect or contain data on household and child characteristics, such as occupational status of women, household wealth data, child illness and vaccination, and maternal and child anthropometry; and c) they did not have corresponding country-level data on agricultural variables or country covariates of interest. If after these exclusions a country still had at least two DHS surveys available for a fixed-effects analysis, those surveys were retained. This resulted in a final sample of 61 DHS surveys from 22 countries between the years 1991 and 2010 (Fig. 1, Table 1).

**Table 1:** Demographic and Health Surveys included in the sample

Country	Survey year (Sample size)
Bangladesh	1997 (2342); 2000 (2617); 2004 (2958)
Benin	1996 (1992); 2001 (2245)
Burkina Faso	1993 (2127); 1999 (2219); 2003 (4148); 2010 (3366)
Cameroon	1998 (1442); 2004 (1668)
Chad	1997 (2781); 2004 (2125)
Colombia	1995 (2289); 2000 (2138); 2005 (5497)
Cote d’Ivoire	1994 (2726); 1999 (864)
Dominican Republic	1991 (1698); 1996 (1842)
Egypt	1992 (3681); 1995 (4704); 2000 (5325); 2003 (3051); 2005 (6334); 2008 (5148)
Ethiopia	2000 (4364); 2005 (1898)
Ghana	1993 (1553); 1998 (1413); 2003 (1598); 2008 (1233)
India	1999 (21,406); 2006 (21,013)
Kenya	1993 (2353); 1998 (2413); 2003 (2298)
Madagascar	1997 (2411); 2004 (2279); 2009 (2549)
Mali	1996 (3667); 2001 (4662); 2006 (5559)
Mozambique	1997 (2645); 2003 (3931)
Nigeria	1999 (1299); 2003 (2306); 2008 (9506)
Senegal	1993 (2212); 2005 (1422)
Tanzania	1992 (3335); 1996 (2729); 2005 (3829); 2010 (3434)
Uganda	1995 (2932); 2001 (2691); 2006 (1258)
Zambia	1992 (2674); 1996 (2954)
Zimbabwe	1994 (1702); 1999 (1403)
Total Sample	212, 258

At the child level, because several countries changed the ages of children included in their surveys over time, we restricted our sample to children aged 6 months to 35 months in order to have a consistent age range across all surveys. The resulting sample of children for the complete case analysis contained 212,258 children (Table 1).

### Measures

#### Policy variables and country-level covariates

##### Nominal rate of assistance to tradable agriculture

The analysis used a value-of-production-weighted average of the NRA for covered tradable products and non-covered tradable products, including export products and import-competing products [52, 55].

##### Country covariates

Covariates at the country level included the NRA for non-tradables, the value of production (VOP) of total agriculture, and the percent of the total VOP of agriculture that was from tradables (exportables or importables). These were derived from the Updated National and Global Estimates of Distortions to Agricultural Incentives, 1955 to 2011 dataset. The NRA for non-tradable products was included to account for the independent effects of policy changes on domestic markets. The VOP of agriculture was included to control for changes in actual agricultural food prices (undistorted) and volumes in order to further isolate the effects of price-distorting agricultural policies. The percent of the VOP of agriculture that was from tradable products (share of tradable agriculture) was included to account for changes in the composition of agriculture and in the importance of tradable agriculture to the economy over time. We deflated VOPs in US dollars using the gross domestic product (GDP) deflator with the year 2000 as the reference year.^1^

In addition, net official development assistance (ODA) and aid received was included from the World Bank’s World Development Indicators, and a governance index indicating level of democracy versus autocracy was included from the Polity IV dataset based on previous research suggesting that these might impact both policy and child health outcomes [57–59].

Several other country-level covariates were considered, including per capita GDP, total population size, percentage of the population that is female, annual rate of growth in the percentage of the population that is rural, population density, percentage of land area that is agricultural, percentage of the population ages 0–14 years, percentage of the population ages 65 and over, labor force participation rate, and overall globalization index from the KOF Index of Globalization. However, due to high variance inflation indicating partial collinearity between these variables and the fixed effects, they were not included in the final models.

#### Individual and household-level variables

##### Nutritional outcomes

Nutrition status in children was measured using height-for-age Z-scores (HAZs), weight-for-height Z-scores (WHZs), and weight-for-age Z-scores (WAZs). According to World Health Organization (WHO) 2006 Child Growth Standards, Z-scores less than − 2 indicate undernutrition in the forms of stunting, wasting, and underweight, respectively. Low HAZ (stunting) reflects long-term undernutrition while low WHZ (wasting) reflects acute undernutrition, and low WAZ (underweight) is a composite measure [3]. The outcomes were analyzed as continuous variables using linear regression.

##### Child, maternal, and household covariates

Characteristics of the child, child’s mother, and child’s household derived from the DHS were also included in the analysis. These were the following: mother’s age, education, total number of children, marital status, and body mass index (BMI); child’s age, sex, months of breastfeeding, recent history of fever, recent history of diarrhea, receipt of vaccinations in his or her first year, and singleton versus multiple birth status; household wealth quintile, rural/urban residence, availability of improved water source, and availability of improved sanitation. These are variables that have been found to be associated with child undernutrition in previous research and have been used as covariates in other recent studies of child undernutrition [60–63]. While some of these variables such as recent history of fever or diarrhea may also be mediators of a relationship between agricultural policies and undernutrition, because of the possibility that they may be affected by other unobserved factors (environmental, ecological, etc.), we have included them in order to produce conservative estimates of our association of interest.

Additionally, a main variable of interest was the occupation of the child’s parent(s). This was divided into four categories: non-agricultural (at least one working parent but no parent employed in agriculture), at least one parent self-employed in agriculture (but none earning wages in agriculture), at least one parent earning wages in agriculture, or all parents unemployed.

### Analysis

Linear regression models were run on the pooled data using Stata versions 13 and 14. Country fixed-effects were included in the models to control for measured and unmeasured time-invariant stable country characteristics, and year fixed-effects were included to control for secular trends due to global processes affecting all countries over time. Thus, models examined only the variation within countries over time. Standard errors were clustered by country to account for non-independence of observations.

For all country-level variables, five-year averages were computed averaging the value of that country-level variable for the concurrent year of the DHS survey with the values of the variable in the four preceding years. This was done to examine the short- to medium-term effects of policy variables, since they may be unlikely to have instantaneous effects on nutrition. In addition, the use of five-year averages for the year of the survey and preceding four years helps to reduce the potential for reverse causation and smooth the country-level indicators.

NRAs and the share of tradable agriculture were divided by 10 so that coefficients corresponded to an increase of 10 percentage points. On average, five-year average NRAs to tradable agriculture changed 17.0 percentage points per country across DHS surveys, with the smallest change being for Mali (0.5 percentage points between 2001 and 2006), the largest change being for Egypt (52.8 percentage points between 1992 and 2005), and the median change being 12.3 percentage points. Thus, there was considerable variation and change in NRAs to tradable agriculture, and a 10-percentage-point change represents a reasonable and observable change at the country level.

The share of tradable agriculture was centered at 50% so that in interaction models, results represented the effect of the NRA and of parental occupation when the share of tradable agriculture was 50%. VOPs, ODA and aid, and other dollar amount variables were entered in the models using the natural log. Models were of the following form:

$$ {Undernutrition}_{itc}={\beta}_0+{\beta}_1{NRA}_{tc}+{\beta}_2{Occupation}_{itc}+{\beta}_3{AgricTrad}_{tc}+{\beta}_4{NRA}_{tc}{Occupation}_{itc}+{\beta}_5{NRA}_{tc}{AgricTrad}_{tc}+{\beta}_6{Occupation}_{itc}{AgricTrad}_{tc}+{\beta}_7{NRA}_{tc}{Occupation}_{itc}{AgricTrad}_{tc}+{\beta}_8{Country}_c+{\beta}_9{Year}_t+{\beta}_{10}{Covariates}_{itc}+{\beta}_{11}{Covariates}_{tc}+{e}_{0 itc} $$

where *i* represents individuals, *t* represents year, and *c* represents country. *Undernutrition*_*itc*_ represents the nutritional outcomes for each child. *NRA*_*tc*_ are the NRAs for each country and year; *Occupation*_*itc*_ represents parental occupation. *AgricTrad*_*tc*_ represents the share of tradable agriculture for each country and year. *NRA*_*tc*_*Occupation*_*itc*_, *NRA*_*tc*_*AgricTrad*_*tc*_, *Occupation*_*itc*_*AgricTrad*_*tc*_, and *NRA*_*tc*_*Occupation*_*itc*_*AgricTrad*_*tc*_ are the interaction terms. *Country*_*c*_ and *Year*_*t*_ are vectors of dummy variables for country and year fixed effects, respectively. *Covariates*_*itc*_ and *Covariates*_*tc*_ are vectors of individual-level and country-level covariates, respectively.

## Results

### Descriptive statistics

Characteristics of the sample are shown in Table 2. On average, children had an HAZ of − 1.64, and 42.1% were stunted. The average WAZ was − 1.15, and 26.4% of children were underweight. Children had an average WHZ of − 0.35, and 12.7% were wasted. There appeared to be positive trends over time for HAZs, while WAZs and WHZs were more variable for the samples (Additional files 1, 2 and 3). Nearly half of the children had at least one parent working in agriculture. Most of those parents were self-employed in agriculture with only 15.4% of all children having any parents earning wages in agriculture. Approximately 2.5% of the children were from households where all parents were unemployed. Children from agricultural households (with a parent self-employed or earning wages in agriculture) had lower HAZs, WAZs, and WHZs and a higher prevalence of stunting, wasting and underweight than children from non-agricultural or unemployed households. They also tended to be of lower socioeconomic status (Table 2).

**Table 2:** Sample characteristics, *N* = 212,258

	Total	Non-agricultural	> 1 parent self-employed agriculture	> 1 parent earning wages agriculture	Parents unemployed	*P*-value^a^
Mean (Std. Dev) / Proportion						
Child’s Characteristics						
Height-for-age Z-score	−1.64 (1.78)	−1.41 (1.76)	− 1.87 (1.74)	−1.83 (1.85)	− 1.51 (1.72)	< 0.001
Child is stunted	42.06%	35.68%	48.57%	47.94%	38.27%	< 0.001
Weight-for-age Z-score^b^	−1.15 (1.45)	−0.90 (1.43)	−1.39 (1.40)	− 1.46 (1.47)	− 0.96 (1.39)	< 0.001
Child is underweight^b^	26.44%	20.72%	31.30%	34.41%	21.18%	< 0.001
Weight-for-height Z-score^c^	−0.35 (1.51)	−0.18 (1.51)	− 0.50 (1.48)	−0.59 (1.53)	− 0.18 (1.49)	< 0.001
Child is wasted^c^	12.71%	10.40%	14.44%	16.53%	10.11%	< 0.001
Child’s sex (Male)	50.60%	50.86%	50.17%	50.63%	51.50%	0.021
Child’s Age (months)	19.85 (8.63)	20.00 (8.65)	19.69 (8.62)	19.93 (8.59)	18.55 (8.61)	< 0.001
Child is a multiple birth	2.16%	2.17%	2.32%	1.81%	1.99%	< 0.001
Child is first born	24.33%	29.47%	17.67%	19.35%	48.58%	< 0.001
Months of breastfeeding	15.24 (7.13)	14.60 (7.16)	15.91 (6.92)	15.93 (7.36)	14.09 (6.73)	< 0.001
Fever in last two weeks	33.50%	31.12%	39.08%	28.09%	35.93%	< 0.001
Diarrhea in last two weeks	21.24%	19.32%	24.25%	20.28%	22.35%	< 0.001
Vaccinations in first year	46.61%	48.68%	47.54%	37.45%	50.82%	< 0.001
Mother’s Characteristics						
Mother’s Age (years)	27.47 (6.51)	27.03 (5.96)	28.18 (7.00)	27.68 (6.76)	24.55 (6.37)	< 0.001
Maternal education (years)	4.16 (4.64)	6.02 (5.07)	2.37 (3.23)	2.20 (3.43)	5.14 (4.37)	< 0.001
Maternal BMI (kg/m^2^)	22.12 (4.29)	23.10 (4.90)	21.21 (3.19)	21.07 (3.67)	22.37 (4.18)	< 0.001
Mother’s total number of children	3.54 (2.40)	3.02 (2.07)	4.16 (2.61)	3.90 (2.51)	2.51 (2.06)	< 0.001
Mother’s marital status						< 0.001
Never married	2.54%	2.15%	1.54%	0.76%	35.39%	
Married	84.09%	84.89%	83.24%	88.29%	54.01%	
Living with partner	8.90%	8.52%	10.35%	7.39%	5.39%	
Widowed	0.93%	0.75%	1.12%	0.99%	1.59%	
Divorced	1.09%	0.96%	1.40%	0.81%	0.13%	
No longer living together	2.44%	2.72%	2.36%	1.76%	2.49%	
Household Characteristics						
Urban	31.54%	54.91%	8.37%	8.59%	44.96%	< 0.001
Wealth quintile						< 0.001
Lowest	22.04%	12.14%	29.41%	37.09%	17.12%	
Second	21.36%	15.67%	26.79%	27.57%	17.22%	
Middle	20.75%	19.43%	23.39%	19.15%	19.77%	
Fourth	19.50%	24.83%	15.16%	12.22%	22.11%	
Highest	16.34%	27.92%	5.25%	3.97%	23.79%	
Improved water	62.73%	78.85%	43.31%	54.75%	70.38%	< 0.001
Improved sanitation	23.75%	38.98%	5.73%	16.11%	27.13%	< 0.001
Parental occupation						
Non-agricultural	47.80%					
At least one parent self-employed in agriculture	34.32%					
At least one parent earning wages in agriculture	15.42%					
Parents unemployed	2.46%					

^a^*P*-values are for categorical variables are from Pearson’s chi-squared test. *P*-values for continuous variables are from Kruskal-Wallis test

^b^ Sample size for weight-for-age Z-scores and underweight is 208,691 children

^c^ Sample size for weight-for-height Z-scores and wasting is 205,556

Five-year averages of the NRA to tradable agriculture for the total sample of country-years included in the analysis ranged from − 72.0 to 45.5% with a mean of − 5.0% and standard deviation of 18.9 percentage points (Table 3). These price distortions generally decreased with time (Additional file 4).

**Table 3:** Descriptive statistics for country-level variables (5-year averages), *N* = 61 surveys (country-years)

Variable	Mean	Std. Dev.	Min	Max
NRA for tradable agricultural products, (% price distortion)	−5.05	18.89	−71.97	45.51
NRA for non-tradable agricultural products (% price distortion)	−0.32	4.76	−22.98	20.54
Share of tradable agriculture (%)	55.28	20.41	16.63	96.97
Per capita GDP PPP	2773.75	2293.01	411.35	8719.08
Total population size	6.93E+ 07	1.88E+ 08	5,781,883	1.13E+ 09
Percent of population that is female	50.04	0.79	48.05	52.26
Annual rate of growth in percentage of the population that is rural	2.05	0.79	0.27	3.73
Percent of population that is rural	67.11	14.18	27.02	88.50
Population density	110.88	209.20	5.57	1045.91
Percent of land area that is agricultural	44.02	20.72	2.70	79.98
Net official development assistance and aid received	1.27E+ 09	1.07E+ 09	8.86E+ 07	5.68E+ 09
Percent of population between ages 0 and 14	43.18	4.79	29.95	49.43
Percent of population ages 65+	3.36	0.76	2.51	5.08
Overall globalization index (KOF)	38.80	8.89	23.10	58.11
Index of governance −10 (strongly autocratic) to + 10 (strongly democratic)	0.73	5.31	−6.8	9
Value of production total agriculture, constant year 2000 USD	8.42E+ 09	1.85E+ 10	3.72E+ 08	1.12E+ 11
Labor force participation rate	70.43	12.92	45.82	89.44

### Fixed-effects regressions

Tables 4, 5 and 6 and Additional files 5, 6 and 7 show the results from the fixed-effects regression models. Model 1 includes the NRA to tradable agriculture; time-varying individual-level and country-level covariates, and country and year fixed-effects. Model 2 adds an interaction between the NRA to tradable agriculture and parental occupation. Model 3 adds interactions with the share of tradable agriculture, including a three-way interaction with the NRA to tradable agriculture and parental occupation.

**Table 4:** Fixed-effects models for HAZs, *n* = 212,258

Height-for-age Z scores	Model 1	Model 2	Model 3
NRA tradable agriculture (10%)	0.02* **(0.00, 0.05)**	*0.02* *(− 0.00, 0.04)*	0.02 (− 0.02, 0.05)
Parental occupation			
Non-agricultural	Ref		
At least one parent self-employed in agriculture	−0.06* **(− 0.12, − 0.01)**	−0.07* **(− 0.12, − 0.01)**	−0.07* **(− 0.13, − 0.01)**
At least one wage-earning parent	− 0.06* **(− 0.11, − 0.00)**	− 0.06** **(− 0.08, − 0.03)**	−0.03 (− 0.09, 0.03)
Parents unemployed	− 0.03 (− 0.09, 0.04)	−0.02 (− 0.08, 0.03)	−0.04 (− 0.09, 0.02)
Share of tradable agriculture (10%)	0.06* **(0.01, 0.10)**	0.06* **(0.01, 0.10)**	0.05* **(0.01, 0.10)**
Parental occupation*NRA tradable agriculture (10%)			
Non-agricultural	Ref		
At least one parent self-employed in agriculture		−0.00 (− 0.03, 0.02)	− 0.02 (− 0.07, 0.02)
At least one wage-earning parent		*0.03* *(− 0.00, 0.07)*	0.06** **(0.03, 0.09)**
Parents unemployed		0.01 (−0.02, 0.03)	0.02 (− 0.02, 0.06)
Share tradable agriculture (10%)*NRA tradable agriculture (10%)			0.00 (−0.01, 0.01)
Parental occupation*Share tradable agriculture (10%)			
Non-agricultural	Ref		
At least one parent self-employed in agriculture			0.01 (−0.02, 0.03)
At least one wage-earning parent			−0.02 (− 0.05, 0.01)
Parents unemployed			0.01 (−0.01, 0.03)
Parental occupation*Share tradable agriculture (10%)*NRA tradable agriculture (10%)			
Non-agricultural	Ref		
At least one parent self-employed in agriculture			0.01 (−0.00, 0.03)
At least one wage-earning parent			−0.02** **(− 0.03, − 0.01)**
Parents unemployed			−0.00 (− 0.02, 0.01)

Notes: Model 1 controls for child’s age, sex, singleton/multiple birth status, birth order, vaccination status, months of breastfeeding, recent fever, and recent diarrhea; mother’s age, number of children, education, BMI, and marital status; household residence (rural/urban), wealth quintile, improved water, and improved sanitation; country-level share of tradable agriculture, log of the value of production of agriculture, NRA for non-tradable agriculture, log of official development assistance and aid, and governance (democratization); country fixed-effects and year fixed-effects. Model 2 adds an interaction between the NRA to tradable agriculture and parental occupation. Model 3 adds interaction terms with the share of tradable agriculture, including a three-way interaction with the NRA to tradable agriculture and parental occupation. Share of tradable agriculture is centered at 50%. Standard errors are clustered by country. *Estimates in italics represent p-values < 0.10.* * represents p-values < 0.05. ** represents p-values < 0.01. *** represents p-values < 0.001

**Table 5:** Fixed-effects models for WAZs, *n* = 208,691

Weight-for-age Z scores	Model 1	Model 2	Model 3
NRA tradable agriculture (10%)	0.05** **(0.02, 0.09)**	0.05** **(0.02, 0.08)**	0.10*** **(0.06, 0.13)**
Parental occupation			
Non-agricultural	Ref		
At least one parent self-employed in agriculture	*−0.05* *(− 0.10, 0.01)*	*− 0.05* *(− 0.11, 0.00)*	*−0.06* *(− 0.13, 0.00)*
At least one wage-earning parent	− 0.06** **(− 0.10, − 0.02)**	−0.06** **(− 0.10, − 0.03)**	−0.03 (− 0.08, 0.01)
Parents unemployed	− 0.01 (− 0.06, 0.05)	−0.01 (− 0.06, 0.05)	−0.02 (− 0.07, 0.03)
Share of tradable agriculture (10%)	0.02 (− 0.04, 0.08)	0.02 (− 0.04, 0.08)	− 0.02 (− 0.08, 0.03)
Parental occupation*NRA tradable agriculture (10%)			
Non-agricultural	Ref		
At least one parent self-employed in agriculture		−0.00 (− 0.03, 0.02)	−0.01 (− 0.05, 0.03)
At least one wage-earning parent		0.04* **(0.01, 0.07)**	0.04** **(0.01, 0.08)**
Parents unemployed		0.01 (−0.01, 0.02)	0.02 (−0.01, 0.06)
Share tradable agriculture (10%)*NRA tradable agriculture (10%)			−0.03*** **(− 0.05, − 0.02)**
Parental occupation*Share tradable agriculture (10%)			
Non-agricultural	Ref		
At least one parent self-employed in agriculture			0.02 (−0.01, 0.05)
At least one wage-earning parent			−0.02 (− 0.06, 0.02)
Parents unemployed			0.01 (−0.00, 0.03)
Parental occupation*Share tradable agriculture (10%)*NRA tradable agriculture (10%)			
Non-agricultural	Ref		
At least one parent self-employed in agriculture			0.00 (−0.01, 0.02)
At least one wage-earning parent			*−0.01* *(− 0.03, 0.00)*
Parents unemployed			−0.01 (− 0.01, 0.01)

Notes: Model 1 controls for child’s age, sex, singleton/multiple birth status, birth order, vaccination status, months of breastfeeding, recent fever, and recent diarrhea; mother’s age, number of children, education, BMI, and marital status; household residence (rural/urban), wealth quintile, improved water, and improved sanitation; country-level share of tradable agriculture, log of the value of production of agriculture, NRA for non-tradable agriculture, log of official development assistance and aid, and governance (democratization); country fixed-effects and year fixed-effects. Model 2 adds an interaction between the NRA to tradable agriculture and parental occupation. Model 3 adds interaction terms with the share of tradable agriculture, including a three-way interaction with the NRA to tradable agriculture and parental occupation. Share of tradable agriculture is centered at 50%. Standard errors are clustered by country. *Estimates in italics represent p-values < 0.10.* * represents p-values < 0.05. ** represents p-values < 0.01. *** represents p-values < 0.001

**Table 6:** Fixed-effects models for WHZs, *n* = 205,556

Weight-for-age Z scores	Model 1	Model 2	Model 3
NRA tradable agriculture (10%)	*0.04* *(−0.01, 0.08)*	*0.04* *(− 0.01, 0.08)*	0.09*** **(0.05, 0.14)**
Parental occupation			
Non-agricultural	Ref		
At least one parent self-employed in agriculture	−0.02 (−0.07, 0.03)	− 0.02 (− 0.07, 0.02)	−0.03 (− 0.09, 0.02)
At least one wage-earning parent	*− 0.04* *(− 0.08, 0.01)*	−0.04 (− 0.09, 0.01)	−0.00 (− 0.04, 0.04)
Parents unemployed	0.01 (− 0.04, 0.05)	0.01 (− 0.04, 0.06)	0.01 (− 0.05, 0.07)
Share of tradable agriculture (10%)	−0.00 (− 0.08, 0.08)	−0.00 (− 0.08, 0.07)	−0.06 (− 0.13, 0.01)
Parental occupation*NRA tradable agriculture (10%)			
Non-agricultural	Ref		
At least one parent self-employed in agriculture		−0.01 (− 0.02, 0.01)	0.01 (− 0.02, 0.03)
At least one wage-earning parent		0.02 (−0.01, 0.05)	*0.03* *(− 0.00, 0.05*)
Parents unemployed		0.01 (−0.01, 0.02)	0.03 (−0.00, 0.06)
Share tradable agriculture (10%)*NRA tradable agriculture (10%)			−0.04*** **(− 0.06, − 0.02)**
Parental occupation*Share tradable agriculture (10%)			
Non-agricultural	Ref		
At least one parent self-employed in agriculture			0.02 (−0.00, 0.04)
At least one wage-earning parent			−0.03 (− 0.07, 0.01)
Parents unemployed			0.00 (−0.02, 0.02)
Parental occupation*Share tradable agriculture (10%)*NRA tradable agriculture (10%)			
Non-agricultural	Ref		
At least one parent self-employed in agriculture			−0.00 (−0.01, 0.01)
At least one wage-earning parent			−0.01 (− 0.03, 0.01)
Parents unemployed			−0.01 (− 0.02, 0.00)

Notes: Model 1 controls for child’s age, sex, singleton/multiple birth status, birth order, vaccination status, months of breastfeeding, recent fever, and recent diarrhea; mother’s age, number of children, education, BMI, and marital status; household residence (rural/urban), wealth quintile, improved water, and improved sanitation; country-level share of tradable agriculture, log of the value of production of agriculture, NRA for non-tradable agriculture, log of official development assistance and aid, and governance (democratization); country fixed-effects and year fixed-effects. Model 2 adds an interaction between the NRA to tradable agriculture and parental occupation. Model 3 adds interaction terms with the share of tradable agriculture, including a three-way interaction with the NRA to tradable agriculture and parental occupation. Share of tradable agriculture is centered at 50%. Standard errors are clustered by country. *Estimates in italics represent p-values < 0.10.* * represents p-values < 0.05. ** represents p-values < 0.01. *** represents p-values < 0.001

Children whose parents were employed in agriculture generally had worse nutritional status (lower HAZs and WAZs) than children from non-agricultural households, even when controlling for other socioeconomic, demographic, and health characteristics. The results showed a positive association between the NRA to tradable agriculture and child nutritional status. Each 10-percentage point increase in the NRA to tradable agriculture was associated with a small but significant increase of 0.02 (95% CI: 95% CI: 0.00–0.05) in HAZs and of 0.05 (95% CI: 0.02–0.09) in WAZs, with a marginally significant increase (*p* < 0.10) of 0.04 (95% CI: -0.01-0.08) for WHZs (Model 1). The effect sizes observed for each 10-percentage point increase in NRA to tradable agriculture were of a similar magnitude to the effect size for each additional year of maternal education on children’s nutritional status (Additional files 5, 6 and 7).

**Fig. 2:**
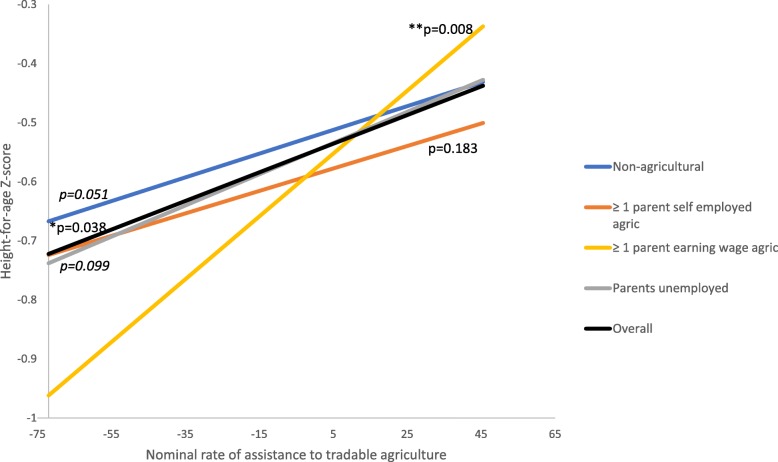
Association between NRA to tradable agriculture and HAZs by parental occupation (two-way interaction model)

**Fig. 3:**
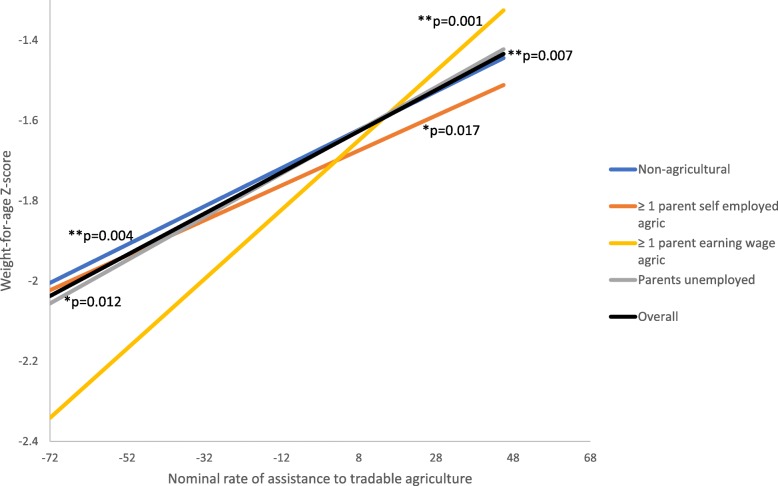
Association between NRA to tradable agriculture and WAZs by parental occupation (two-way interaction model)

**Fig. 4:**
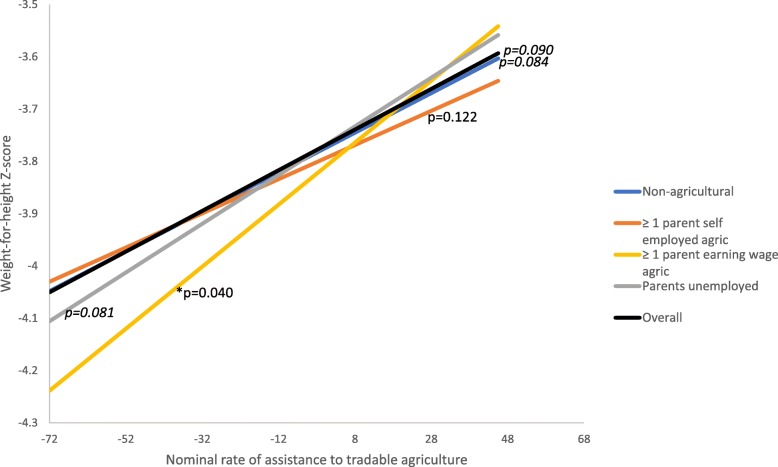
Association between NRA to tradable agriculture and WHZs by parental occupation (two-way interaction model)

When examining whether the association between the NRA to tradable agriculture differs for children according to the occupational status of their parents, some interaction effects were observed (Model 2). The positive association that was observed between the NRA to tradable agriculture and nutritional status in the overall sample was still present among children from non-agricultural households for WAZs and marginally significant for HAZs and WHZs. However, the association was more positive (stronger) for children who had at least one parent earning wages in agriculture. Figures 2, 3 and 4 show the associations between NRA to tradable agriculture and HAZs, WAZs, and WHZs, respectively for the overall sample (from Model 1) and by parental occupation based on the two-way interactions in Model 2 when all covariates are set to the reference group or zero. The figures illustrate that the positive associations between the NRA to tradable agriculture and child nutritional status are strongest for children with a parent earning wages in agriculture, as indicated by the slopes and their *p*-values. The interaction models showed that the difference in association for children with at least one wage-earning agricultural parent compared to children from non-agricultural households was marginally significant for HAZs (coefficient = 0.03, *p*-value = 0.06), statistically significant for WAZs (coefficient = 0.04, *p* = 0.01), but not significant for WHZs (coefficient = 0.02, *p* = 0.12) (Tables 4, 5 and 6, Model 2).

Effects of assistance to tradable agriculture on nutrition may also differ based on the level of trade in a country’s agricultural sector. To assess this, in Model 3, we incorporated interactions with the share of tradable agriculture and observed significant interactions for WAZs and WHZs. As the share of tradable agriculture increased, the beneficial effect of increases in the NRA to tradable agriculture on WAZs and WHZs decreased.

### Sensitivity analyses

In sensitivity analyses using country-level variables from only the year of the DHS survey rather than five-year averages, the positive coefficient for NRA to tradable agriculture was no longer statistically significant in main effects models for the overall sample or among children of non-agricultural parents. However, for HAZs and WAZs there was still a significant positive interaction among children who had at least one parent working in wage agriculture compared to children from non-agricultural households. Thus, though some results were less pronounced, there were overall similarities to our main results even when using short-term or concurrent NRA values.

Sensitivity analyses adding the square of child’s age to examine potential nonlinear relationships also produced similar results to those presented here, with some associations for our variables of interest being even stronger.. Analyses examining only African countries, and thus excluding large samples such as India, also produced similar results to those reported here.

Sensitivity analyses were also conducted using the dichotomous undernutrition outcomes of stunting, underweight, and wasting based on HAZs, WAZs, and WHZs, less than − 2, respectively. These logistic models again produced similar results, showing reduced odds of stunting (marginally significant; OR: 0.97, 95% CI: 0.935–1.006) and underweight (OR: 0.96, 95% CI: 0.930–0.996) with increases in the NRA to tradable agriculture in main effects models for the overall sample, and a greater reduction in odds of stunting (OR: 0.96, 95% CI: 0.92–1.00), underweight (OR: 0.93, 95% CI: 0.91–0.96), and wasting (OR: 0.93, 95% CI: 0.90–0.96) among children with a parent earning wages in agriculture compared to children from non-agricultural households in interaction models. Finally, in sensitivity analyses using mixed-effects models (random effects for survey and country) rather than country fixed effects, and controlling for all the additional time-varying country-level covariates initially considered (see Methods and Table 3), we continued to observe significant increases in nutritional Z-scores with increases in the NRA to tradable agriculture and a significant interaction for children with a parent earning wages in agriculture compared to those from non-agricultural households.

## Discussion

In fixed-effects analyses, we observed a small but significant positive association between increases in rates of assistance to tradable agriculture and child nutritional status, particularly for WAZs. The magnitude of these associations was greatest among children who had a parent earning wages in agriculture. We also observed in interactions that the association between the NRA and child WAZs and WHZs diminished as the share of tradable agriculture in the value of production increased.

On average, children from agricultural households had worse nutritional status than those from non-agricultural households. This result, as well as the associations between child nutritional status and household wealth and urban location (Additional files 5, 6 and 7), indicate that socioeconomic health disparities remain a significant problem.

Similar to our findings, Webb and Block [54] observed in their cross-country study that declines in stunting prevalence as rural population shares decreased were more rapid among countries supporting agriculture (positive relative rates of assistance) than among those that were not. Support for agriculture indicated by positive rates of assistance, sometimes occurs in the form of trade protection such as through import tariffs or export subsidies. These would result in increased consumer food prices which one might expect to lead to reduced food security and nutritional status. However, there have been some indications that while increases in food prices predict greater poverty in the short run in microeconomic (household) models in LMICs, macroeconomic models allowing changes in agricultural supply and wages often predict reductions in poverty. Headey [64] observed that although food price increases may be detrimental to poverty in the short run, increased food prices result in reductions in poverty and inequality in the long run (one to five years), as higher prices are passed on to producers. This suggests the importance of income, which Shankar [28] suggests may be the most important pathway linking trade and diet.

However, other studies examining links between macroeconomic factors and nutrition have instead found trade liberalization, and not trade protections, to be associated with improved nutrition. Atkin [65] argued that liberalized trade increases consumption and improves the nutrition of the poor, but this may only occur in the long run, when preferences change to favor lower-priced imported food. In their dynamic panel analysis, Dithmer and Abdulai [49] found that trade openness—as measured by trade volumes as a percentage of GDP, reductions in tariff rates, or globalization—was associated with improved dietary consumption, quality, and diversity. Nandi and colleagues [66] similarly observed that trade liberalization as measured by lower mean tariff rates, was associated with decreased odds of underweight compared to normal weight. These studies suggest that agricultural assistance in the form of import tariffs may not be beneficial for undernutrition but detrimental.

In the context of our findings, this may imply that improvements in nutrition due to increases in the NRA are more likely to result from agricultural support in the form of reduced taxation on exports (liberalization) rather than from increased protection. Supporting this hypothesis, Olper and colleagues [67] observed greater reductions in child mortality in trade reforming countries that had larger reductions in taxation of agriculture. As previously mentioned, in the present sample during the time period studied, many countries had mostly negative NRAs due to taxation of exports, which decreased toward zero over time, becoming more liberalized. Effects of increases in NRAs on nutrition in this case might therefore reflect this mechanism of liberalization through reduced taxation. Indeed, a check of this by interacting the NRA to tradable agriculture with an indicator for positive NRAs confirmed that increases in the NRA to tradable agriculture were associated with higher HAZs and WAZs when the NRAs were negative (and increases therefore reflected reduced taxation and liberalization), but not when they were positive (where increases implied further price distortions).

If the associations observed in our study are causal, the results would imply that government assistance to tradable agriculture, through measures such as tax reduction, results in minor improvements to nutritional status, particularly for children whose parents earn wages in agriculture. Given that children from agricultural households tended to be more likely to be undernourished, this would suggest that policies that result in increased assistance to tradable agriculture could in theory contribute to reducing disparities in undernutrition. However, as a country’s agriculture sector becomes more integrated in the global economy (the share of tradable agriculture increases), government assistance to tradable agriculture may become less relevant.

Our result of slight increases in nutritional Z-scores, particularly WAZs, in the overall sample, along with greater increases among children with a parent earning wages in agriculture, could imply that the positive effects of government assistance to tradable agriculture are potentially operating on nutritional outcomes by contributing to overall improvements in the economy, as well as through additional positive income effects (higher returns or earnings) for households employed in wage-earning agriculture. It should also be noted, however, that in many of these countries the agricultural population is decreasing with time while urban populations are increasing. One might therefore expect that the impacts observed here may become less pronounced as economies shift away from agriculture as well.

### Alternative explanations

It is possible that these results were confounded by other simultaneous changes that accompany changes in the NRA to tradable agriculture. Although the use of country and year fixed-effects controlled for time-constant country-level differences that may be confounding the results, and for global trends, respectively, they cannot account for time-varying country-level confounders that may cause both changes in policy and changes in nutrition. We attempted to control for some such factors by including covariates such as the VOP of agriculture, the NRA for non-tradable agriculture, the share of tradable agriculture, ODA and aid, and governance (democratization). Nevertheless, there could be other policy and non-policy variables confounding the results. For example, NRAs are correlated with factors such as GDP per capita [50]. Due to partial collinearity between several time-varying country-level variables and the fixed effects, the results presented here do not report estimates for many of the country-level covariates considered, since estimates for these variables would be unreliable and potentially unstable. However, even in models including controls for variables such as log per capita GDP, annual rate of growth in percentage of the population that is rural, population density, percentage of land area that is agricultural, proportion of the population that is agricultural, labor force participation rate, and globalization index, et cetera, our main results were qualitatively similar. There was still a small but significant positive association between the NRA to tradable agriculture and nutritional status which was more pronounced among children whose parents earned wages in agriculture. Still, the possibility of endogeneity or residual time-varying confounding by unobserved variables remains because of the observational nature of the study. Factors such as climate shocks (flood, drought, etc.) and conflict may be related to changes in government rates of assistance for instance, and it is possible that countries that increase assistance to agriculture are also more likely to enact redistribution policies that may impact nutrition. These were not accounted for in this analysis and future studies may seek to expand on this work by attempting to capture such data.

### A note on overnutrition

It is important to note that many LMICs are facing a “double burden of malnutrition” with persistent undernutrition alongside an increasing burden of overnutrition in the form of overweight and obesity. A growing body of research suggests that trade liberalization and agricultural policies may also be contributing to the rise in overnutrition [41, 42, 45–47, 66]. While an explicit examination of overnutrition was beyond the aims of this paper, it is an area that deserves further attention. Given that our results showed increases in Z-scores with increased assistance to tradable agriculture, which during this time period was largely in the form of reduced taxation, it is plausible that such policy changes are also associated with overnutrition.

### Limitations

There are some limitations to this study. This analysis does not take into account the effect of trade policies of other countries (particularly high-income countries) on welfare and nutrition of children in the sample. While LMICs generally liberalized prices for agriculture from the 1980s to 2000s, particularly through reducing taxation on exports, there was little change or reduction in OECD countries’ support or subsidization of agricultural producers during the same period, though the nature of support has changed. Full liberalization, particularly of high-income-country agriculture, is expected to have net benefits to LMICs; however, such progress may be stalled with the failure of the Doha Rounds [25, 28, 30, 53]. While we did not account for high-income country policies in our models, our use of time-fixed effects should account for changes in global trade policies, though not bilateral or multilateral agreements.

Although the interaction between the NRA and parental occupation is suggestive of income pathways as a main mechanism linking agricultural trade policy and nutrition in these countries, our analysis was unable to directly examine income and consumption pathways. The DHS data do not include income or consumption data; therefore, we could not look at the association between the agricultural policies and incomes or consumption among different types of households (agricultural, non-agricultural) to link them with child nutritional outcomes. Other datasets such as the Living Standards Measurement Surveys may enable one to link policy data to incomes and consumption and help to illuminate these potential pathways. However, these surveys typically do not include measurements of health status. Qualitative research examining various case studies of globalization and policy change, such as demonstrated by Brown and Labonté [68], may be a useful way to understand the pathways through which these policies can impact nutrition. Further work using such methods would benefit this field of research.

It is possible that several covariates used in the analysis (recent history of fever or diarrhea, share of tradable agriculture, total VOP of agriculture, etc.) may actually mediate the relationship between agricultural trade policies and child undernutrition. By including them in our models, we may therefore be attenuating our effect of interest. However, because such variables could in theory also change as a result of simultaneous changes in other unobserved factors, we felt it necessary to control for them to attempt to isolate the effects of our policy variable as best as possible. Therefore, it is likely that our results represent conservative estimates of the associations between NRAs to tradable agriculture and child nutritional status. Additionally, it should be noted that children with a parent missing occupational information were classified according to the parent for whom occupation was given. Thus, if some of these children receive support from another parent who is employed in a different sector, they may have been misclassified.

The relevant etiological period for policy effects on child nutrition may also be unknown. We examined effects of policy measures averaged over the year of the survey and the preceding four years, and we simultaneously controlled for the age of the child. We also explored potentially instantaneous effects in sensitivity analyses using country-level variables from only the year of the survey. However, future analyses may wish to explore other time lags or potential effects of policies in the year of birth of the child, such that the each child in a survey may have a different policy exposure. On another note, it has been observed that malnutrition in young children tends to be unresponsive to economic growth [63]. Therefore, results among our sample which includes children ages 6 to 35 months could actually be more conservative than what might be observed in an older sample of children.

## Conclusions

Our cross-national analysis shows that socioeconomic disparities in child nutrition remain a significant issue to be addressed. We observed that government support to tradable agriculture through measures such as reduced taxation may be associated with small increases in child nutritional status, particularly for children with parents earning wages in agriculture. Such interventions, if causal, could therefore contribute to reducing disparities in undernutrition. However, as agricultural markets for countries become more globalized or less dominated by non-tradable staple crops, such interventions may become less important. Understanding how agricultural and trade policies impact undernutrition may help to promote health and development and reduce inequality worldwide. Nevertheless, such impacts may be modest and are just one of several interventions needed to improve nutrition.

## Additional files

Additional file 1: Mean HAZs of survey samples by country and year (DOCX 21 kb)

Additional file 2: Mean WAZs of survey samples by country and year (DOCX 21 kb)

Additional file 3: Mean WHZs of survey samples by country and year (DOCX 21 kb)

Additional file 4: Five-year average NRAs to tradable agriculture by country and year (DOCX 21 kb)

Additional file 5: Full Fixed-Effects Models for HAZs, *n*=212,258 (DOCX 18 kb)

Additional file 6: Full Fixed-Effects Models for WAZs, *n*=208,691 (DOCX 18 kb)

Additional file 7: Full Fixed-Effects Models for WHZs, *n* = 205,556 (DOCX 17 kb)

## Abbreviations

BMI: Body Mass Index
DHS: Demographic and health survey
GDP: Gross domestic product
HAZs: Height-for-age Z-scores
LMICs: Low- and middle-income countries
NRA: Nominal rate of assistance to agriculture
ODA: Official development assistance
PPP: Purchasing power parity
SAPs: Structural Adjustment Programs
VOP: Value of production
WAZs: Weight-for-age Z-scores
WHO: World Health Organization
WHZs: Weight-for-height Z-scores

## Footnotes

1 For years after 2005, VOPs for non-covered products did not include a breakdown by importables, exportables, and non-tradables. Therefore, we estimated the share of VOPs for non-covered importables and exportables for the years 2006 onward by assuming the same proportions of importables, exportables, and non-tradables among non-covered products as in previous years. These proportions were then multiplied by the total VOP for non-covered products to estimate VOPs by product type, and these estimates were used in calculations of share of tradable agriculture.
