# Correction to: Agricultural trade policies and child nutrition in low- and middle-income countries: a cross-national analysis

**DOI:** 10.1186/s12992-019-0471-0

**Published:** 2019-04-10

**Authors:** Kafui Adjaye-Gbewonyo, Sebastian Vollmer, Mauricio Avendano, Kenneth Harttgen

**Affiliations:** 1 Innovative Methods & Metrics for Agriculture and Nutrition Actions, Tufts University Friedman School of Nutrition Science and Policy, 150 Harrison Avenue, Boston, MA 02111 USA; 2 Institute of Advanced Studies, University College London, Gower Street, London, WC1E 6BT UK; 3 Harvard T. H. Chan School of Public Health, 677 Huntington Avenue, Boston, MA 02115 USA; 4 University of Göttingen; Center for Modern Indian Studies, Waldweg 26, Altbau 1.118, 37073 Göttingen, Germany; 5 Department of Global Health and Social Medicine, King’s College London, Strand Campus, Strand, London, WC2R2LS UK; 6 ETH Zürich, NADEL Center for Development and Cooperation, Clausiusstrasse 37, 8092 Zurich, Switzerland

**Correction to:** ***Globalization and Health (*** **2019) 15:21**

**DOI: 10.1186/s12992-019-0463-0**

Following publication of the original article [1], the author flagged that two sets of errors had unfortunately been missed during production of the article.

Firstly, in the ‘Results’ of the article’s Abstract it read “**(0.02, 95% CI,0.00–0.05**)”, while it should read “**(0.02, 95% CI: 0.00–0.05)**”.

Secondly, in Table 2 of the article, in the 4th and 5th columns, it read “> 1 parent self-employed agriculture” and “> 1 parent earning wages agriculture”, respectively. However, the “>” symbol in these two columns should instead be “**≥**”, to signify ‘1 or more parents’, rather than ‘more than 1 parent’.

The publisher apologizes for this processing error.
