# Supplementary material for: Agricultural trade policies and child nutrition in low- and middle-income countries: a cross-national analysis
Source: Global Health. 2019 Mar 15;15:21. doi: 10.1186/s12992-019-0463-0 (PMC6420724; doi:10.1186/s12992-019-0463-0)
Supplement: Supplementary file 1 — Mean HAZs of survey samples by country and year (DOCX 21 kb) [file 12992_2019_463_MOESM1_ESM.docx]

Additional File 1. Mean HAZs of survey samples by country and year

|  |
| --- |
|  |
