# Supplementary material for: Agricultural trade policies and child nutrition in low- and middle-income countries: a cross-national analysis
Source: Global Health. 2019 Mar 15;15:21. doi: 10.1186/s12992-019-0463-0 (PMC6420724; doi:10.1186/s12992-019-0463-0)
Supplement: Supplementary file 6 — Full Fixed-Effects Models for WAZs, n=208,691 (DOCX 18 kb) [file 12992_2019_463_MOESM6_ESM.docx]

**Additional File 6. Full Fixed-Effects Models for WAZs, n=208,691**

| **Weight-for-age Z scores** | **Model 0** | **Model 1** | **Model 2** | **Model 3** |
| --- | --- | --- | --- | --- |
| ***Child characteristics*** |  |  |  |  |
| *Child's age (months)* | **-0.00***  **(-0.01, -0.00)** | **-0.00***  **(-0.01, -0.00)** | **-0.00***  **(-0.01, -0.00)** | **-0.00***  **(-0.01, -0.00)** |
| *Child's sex* | |  |  |  |
| Male | Ref |  |  |  |
| Female | **0.13*****  **(0.09, 0.18)** | **0.13*****  **(0.09, 0.18)** | **0.13*****  **(0.09, 0.18)** | **0.13*****  **(0.09, 0.18)** |
| *Multiple birth* | |  |  |  |
| No | Ref |  |  |  |
| Yes | **-0.55*****  **(-0.71, -0.39)** | **-0.55*****  **(-0.71, -0.40)** | **-0.55*****  **(-0.71, -0.40)** | **-0.55*****  **(-0.71, -0.40)** |
| *First-born child* | |  |  |  |
| No | Ref |  |  |  |
| Yes | **0.05***  **(0.01, 0.09)** | **0.05***  **(0.01, 0.09)** | **0.05***  **(0.01, 0.09)** | **0.05***  **(0.01, 0.09)** |
| *Vaccinated in first year* |  |  |  |  |
| No | Ref |  |  |  |
| Yes | **0.06****  **(0.02, 0.10)** | **0.06****  **(0.02, 0.10)** | **0.06****  **(0.02, 0.10)** | **0.06****  **(0.02, 0.09)** |
| *Diarrhoea in past two weeks* | |  |  |  |
| No | Ref |  |  |  |
| Yes | **-0.17*****  **(-0.22, -0.13)** | **-0.17*****  **(-0.22, -0.13)** | **-0.17*****  **(-0.22, -0.13)** | **-0.17*****  **(-0.22, -0.12)** |
| *Fever in the past two weeks* |  |  |  |  |
| No | Ref |  |  |  |
| Yes | **-0.14*****  **(-0.18, -0.11)** | **-0.14*****  **(-0.18, -0.11)** | **-0.14*****  **(-0.18, -0.11)** | **-0.14*****  **(-0.18, -0.11)** |
| *Months of breastfeeding* | **-0.02*****  **(-0.02, -0.01)** | **-0.02*****  **(-0.02, -0.01)** | **-0.02*****  **(-0.02, -0.01)** | **-0.02*****  **(-0.02, -0.01)** |
| ***Maternal Characteristics*** |  |  |  |  |
| *Mother's age* | **0.01*****  **(0.01, 0.02)** | **0.01*****  **(0.01, 0.02)** | **0.01*****  **(0.01, 0.02)** | **0.01*****  **(0.01, 0.02)** |
| *Mother's total number of children* | **-0.04*****  **(-0.05, -0.02)** | **-0.04*****  **(-0.05, -0.02)** | **-0.04*****  **(-0.05, -0.02)** | **-0.04*****  **(-0.05, -0.02)** |
| *Maternal education (years)* | **0.03****  **(0.01, 0.05)** | **0.03****  **(0.01, 0.05)** | **0.03****  **(0.01, 0.05)** | **0.03****  **(0.01, 0.05)** |
| *Mother's BMI* | **0.06*****  **(0.04, 0.07)** | **0.06*****  **(0.04, 0.07)** | **0.06*****  **(0.04, 0.07)** | **0.06*****  **(0.04, 0.07)** |
| *Mother’s Marital status* | |  |  |  |
| Never married | Ref |  |  |  |
| Married | **0.08***  **(0.01, 0.15)** | **0.07***  **(0.00, 0.14)** | *0.07*  *(-0.00, 0.15)* | **0.07***  **(0.00, 0.14)** |
| Living with partner | **0.08***  **(0.02, 0.15)** | **0.08***  **(0.02, 0.15)** | **0.08***  **(0.01, 0.15)** | **0.08***  **(0.02, 0.14)** |
| Widowed | **0.09***  **(0.01, 0.17)** | **0.09***  **(0.01, 0.17)** | **0.09***  **(0.00, 0.17)** | **0.08***  **(0.01, 0.16)** |
| Divorced | 0.00  (-0.08, 0.09) | 0.00  (-0.08, 0.08) | -0.00  (-0.09, 0.08) | -0.00  (-0.08, 0.08) |
| No longer living together/separated | 0.05  (-0.03, 0.12) | 0.05  (-0.03, 0.12) | 0.04  (-0.03, 0.12) | 0.05  (-0.03, 0.12) |
| ***Household characteristics*** |  |  |  |  |
| *Residence* | |  |  |  |
| Rural | Ref |  |  |  |
| Urban | 0.01  (-0.03, 0.05) | 0.01  (-0.03, 0.04) | 0.01  (-0.03, 0.04) | 0.00  (-0.03, 0.04) |
| *Wealth quintile* | |  |  |  |
| Lowest | Ref |  |  |  |
| Second | **0.05***  **(0.01, 0.09)** | **0.05***  **(0.01, 0.09)** | **0.05***  **(0.01, 0.09)** | **0.05***  **(0.01, 0.09)** |
| Middle | **0.11****  **(0.04, 0.17)** | **0.11****  **(0.05, 0.17)** | **0.11****  **(0.05, 0.17)** | **0.11****  **(0.05, 0.17)** |
| Fourth | **0.16*****  **(0.10, 0.22)** | **0.16*****  **(0.10, 0.22)** | **0.16*****  **(0.10, 0.22)** | **0.16*****  **(0.10, 0.21)** |
| Highest | **0.25*****  **(0.20, 0.30)** | **0.25*****  **(0.20, 0.30)** | **0.25*****  **(0.20, 0.29)** | **0.25*****  **(0.20, 0.29)** |
| *Parental occupation* |  |  |  |  |
| Non-agricultural | Ref |  |  |  |
| At least one parent self-employed in agriculture | -0.04  (-0.10, 0.01) | *-0.05*  *(-0.10, 0.01)* | *-0.05*  *(-0.11, 0.00)* | *-0.06*  *(-0.13, 0.00)* |
| At least one wage-earning parent | **-0.07****  **(-0.11, -0.03)** | **-0.06****  **(-0.10, -0.02)** | **-0.06****  **(-0.10, -0.03)** | -0.03  (-0.08, 0.01) |
| Parents unemployed | -0.01  (-0.06, 0.05) | -0.01  (-0.06, 0.05) | -0.01  (-0.06, 0.05) | -0.02  (-0.07, 0.03) |
| *Improved water* | |  |  |  |
| No | Ref |  |  |  |
| Yes | 0.02  (-0.03, 0.07) | 0.02  (-0.04, 0.07) | 0.01  (-0.04, 0.06) | 0.01  (-0.04, 0.06) |
|  |  |  |  |  |
| *Improved sanitation* | | |  |  |
| No | Ref |  |  |  |
| Yes | 0.05  (-0.03, 0.14) | 0.06  (-0.02, 0.14) | 0.06  (-0.02, 0.14) | *0.07*  *(-0.01, 0.15)* |
| **Country-level variables** |  |  |  |  |
| *NRA tradable agriculture (10%)* | **0.04*****  **(0.02, 0.06)** | **0.05****  **(0.02, 0.09)** | **0.05****  **(0.02, 0.08)** | **0.10*****  **(0.06, 0.13)** |
| *Share of tradable agriculture (10%)* |  | 0.02  (-0.04, 0.08) | 0.02  (-0.04, 0.08) | -0.02  (-0.08, 0.03) |
| *Log value of production of agriculture* |  | 0.00  (-0.14, 0.14) | 0.02  (-0.12, 0.16) | -0.06  (-0.15, 0.03) |
| *NRA non-tradable agriculture (10%)* |  | -0.04  (-0.13, 0.05) | -0.03  (-0.11, 0.06) | -0.03  (-0.11, 0.05) |
| *Log Official development assistance & aid* |  | **-0.08****  **(-0.14, -0.03)** | **-0.08****  **(-0.13, -0.03)** | **-0.05***  **(-0.09, -0.01)** |
| *Governance (democratization)* |  | 0.02  (-0.01, 0.05) | 0.02  (-0.00, 0.05) | **0.05*****  **(0.03, 0.07)** |
| ***Interactions*** |  |  |  |  |
| *Parental occupation*NRA tradable agriculture (10%)* |  |  |  |  |
| Non-agricultural | Ref |  |  |  |
| At least one parent self-employed in agriculture |  |  | -0.00  (-0.03, 0.02) | -0.01  (-0.05, 0.03) |
| At least one wage-earning parent |  |  | **0.04***  **(0.01, 0.07)** | **0.04****  **(0.01, 0.08)** |
| Parents unemployed |  |  | 0.01  (-0.01, 0.02) | 0.02  (-0.01, 0.06) |
| *Share tradable agriculture (10%)*NRA tradable agriculture (10%)* |  |  |  | **-0.03*****  **(-0.05, -0.02)** |
| *Parental occupation*Share tradable agriculture (10%)* |  |  |  |  |
| Non-agricultural | Ref |  |  |  |
| At least one parent self-employed in agriculture |  |  |  | 0.02  (-0.01, 0.05) |
| At least one wage-earning parent |  |  |  | -0.02  (-0.06, 0.02) |
| Parents unemployed |  |  |  | 0.01  (-0.00, 0.03) |
| *Parental occupation*Share tradable agriculture (10%)*NRA tradable agriculture (10%)* |  |  |  |  |
| Non-agricultural | Ref |  |  |  |
| At least one parent self-employed in agriculture |  |  |  | 0.01  (-0.01, 0.02) |
| At least one wage-earning parent |  |  |  | *-0.01*  *(-0.03, 0.00)* |
| Parents unemployed |  |  |  | -0.01  (-0.02, 0.01) |

Notes: Share of tradable agriculture is centered at 50%. Standard errors clustered by country. *Estimates in italics represent p-values < 0.10.* *** represents p-values <0.05. ** represents p-values < 0.01. *** represents p-values < 0.001.**
