# Supplementary material for: Agricultural trade policies and child nutrition in low- and middle-income countries: a cross-national analysis
Source: Global Health. 2019 Mar 15;15:21. doi: 10.1186/s12992-019-0463-0 (PMC6420724; doi:10.1186/s12992-019-0463-0)
Supplement: Supplementary file 7 — Full Fixed-Effects Models for WHZs, n = 205,556 (DOCX 17 kb) [file 12992_2019_463_MOESM7_ESM.docx]

**Additional File 7. Full Fixed-Effects Models for WHZs, n=205,556**

| **Weight-for-age Z scores** | **Model 0** | **Model 1** | **Model 2** | **Model 3** |
| --- | --- | --- | --- | --- |
| ***Child characteristics*** |  |  |  |  |
| *Child's age (months)* | **0.02*****  **(0.01, 0.02)** | **0.02*****  **(0.01, 0.02)** | **0.02*****  **(0.01, 0.02)** | **0.02*****  **(0.01, 0.02)** |
| *Child's sex* | |  |  |  |
| Male | Ref |  |  |  |
| Female | **0.08*****  **(0.07, 0.10)** | **0.08*****  **(0.07, 0.10)** | **0.08*****  **(0.07, 0.10)** | **0.08*****  **(0.07, 0.10)** |
| *Multiple birth* | |  |  |  |
| No | Ref |  |  |  |
| Yes | **-0.30*****  **(-0.39, -0.20)** | **-0.30*****  **(-0.39, -0.20)** | **-0.30*****  **(-0.39, -0.20)** | **-0.30*****  **(-0.39, -0.20)** |
| *First-born child* | |  |  |  |
| No | Ref |  |  |  |
| Yes | **0.03***  **(0.00, 0.05)** | **0.03***  **(0.00, 0.05)** | **0.03***  **(0.00, 0.05)** | **0.03***  **(0.00, 0.05)** |
| *Vaccinated in first year* |  |  |  |  |
| No | Ref |  |  |  |
| Yes | **0.06*****  **(0.03, 0.09)** | **0.06*****  **(0.03, 0.09)** | **0.06*****  **(0.03, 0.09)** | **0.05****  **(0.03, 0.08)** |
| *Diarrhoea in past two weeks* | |  |  |  |
| No | Ref |  |  |  |
| Yes | **-0.15*****  **(-0.19, -0.11)** | **-0.15*****  **(-0.18, -0.11)** | **-0.15*****  **(-0.18, -0.11)** | **-0.14*****  **(-0.18, -0.11)** |
| *Fever in the past two weeks* |  |  |  |  |
| No | Ref |  |  |  |
| Yes | **-0.18*****  **(-0.21, -0.15)** | **-0.18*****  **(-0.21, -0.15)** | **-0.18*****  **(-0.21, -0.15)** | **-0.18*****  **(-0.21, -0.15)** |
| *Months of breastfeeding* | **-0.01*****  **(-0.01, -0.00)** | **-0.01*****  **(-0.01, -0.00)** | **-0.01*****  **(-0.01, -0.00)** | **-0.01*****  **(-0.01, -0.00)** |
| ***Maternal Characteristics*** |  |  |  |  |
| *Mother's age* | -0.00  (-0.01, 0.00) | -0.00  (-0.01, 0.00) | -0.00  (-0.01, 0.00) | -0.00  (-0.01, 0.00) |
| *Mother's total number of children* | **-0.01****  **(-0.02, -0.00)** | **-0.01***  **(-0.02, -0.00)** | **-0.01***  **(-0.02, -0.00)** | **-0.01****  **(-0.02, -0.00)** |
| *Maternal education (years)* | **0.02*****  **(0.01, 0.03)** | **0.02*****  **(0.01, 0.03)** | **0.02*****  **(0.01, 0.03)** | **0.02*****  **(0.01, 0.03)** |
| *Mother's BMI* | **0.05*****  **(0.04, 0.07)** | **0.05*****  **(0.04, 0.07)** | **0.05*****  **(0.04, 0.07)** | **0.05*****  **(0.04, 0.07)** |
| *Mother’s Marital status* | |  |  |  |
| Never married | Ref |  |  |  |
| Married | 0.02  (-0.04, 0.07) | 0.02  (-0.04, 0.07) | 0.01  (-0.04, 0.07) | 0.02  (-0.04, 0.07) |
| Living with partner | 0.01  (-0.04, 0.07) | 0.01  (-0.05, 0.07) | 0.01  (-0.05, 0.07) | 0.01  (-0.04, 0.07) |
| Widowed | -0.01  (-0.09, 0.07) | -0.01  (-0.09, 0.07) | -0.01  (-0.09, 0.07) | -0.01  (-0.09, 0.07) |
| Divorced | -0.07  (-0.16, 0.02) | -0.07  (-0.16, 0.02) | *-0.07*  *(-0.16, 0.01)* | -0.07  (-0.16, 0.02) |
| No longer living together/separated | 0.03  (-0.04, 0.10) | 0.03  (-0.04, 0.09) | 0.02  (-0.04, 0.09) | 0.02  (-0.04, 0.09) |
| ***Household characteristics*** |  |  |  |  |
| *Residence* | |  |  |  |
| Rural | Ref |  |  |  |
| Urban | **-0.04***  **(-0.07, -0.00)** | **-0.04***  **(-0.07, -0.01)** | **-0.04***  **(-0.07, -0.01)** | **-0.04***  **(-0.08, -0.01)** |
| *Wealth quintile* | |  |  |  |
| Lowest | Ref |  |  |  |
| Second | **0.04***  **(0.00, 0.07)** | **0.04***  **(0.00, 0.07)** | **0.04***  **(0.00, 0.07)** | **0.04***  **(0.00, 0.07)** |
| Middle | **0.08****  **(0.03, 0.13)** | **0.08****  **(0.03, 0.13)** | **0.08****  **(0.03, 0.13)** | **0.08****  **(0.03, 0.12)** |
| Fourth | **0.10*****  **(0.06, 0.14)** | **0.10*****  **(0.06, 0.14)** | **0.10*****  **(0.06, 0.14)** | **0.10*****  **(0.06, 0.14)** |
| Highest | **0.13*****  **(0.09, 0.17)** | **0.13*****  **(0.09, 0.17)** | **0.13*****  **(0.09, 0.16)** | **0.13*****  **(0.09, 0.16)** |
| *Parental occupation* |  |  |  |  |
| Non-agricultural | Ref |  |  |  |
| At least one parent self-employed in agriculture | -0.02  (-0.07, 0.03) | -0.02  (-0.07, 0.03) | -0.02  (-0.07, 0.02) | -0.03  (-0.09, 0.02) |
| At least one wage-earning parent | *-0.04*  *(-0.08, 0.00)* | *-0.04*  *(-0.08, 0.01)* | -0.04  (-0.09, 0.01) | -0.00  (-0.04, 0.04) |
| Parents unemployed | 0.00  (-0.04, 0.05) | 0.01  (-0.04, 0.05) | 0.01  (-0.04, 0.06) | 0.01  (-0.05, 0.07) |
| *Improved water* | |  |  |  |
| No | Ref |  |  |  |
| Yes | 0.02  (-0.01, 0.04) | 0.02  (-0.01, 0.04) | 0.01  (-0.01, 0.04) | 0.01  (-0.01, 0.04) |
| *Improved sanitation* | | |  |  |
| No | Ref |  |  |  |
| Yes | -0.00  (-0.08, 0.08) | 0.00  (-0.08, 0.08) | 0.00  (-0.07, 0.08) | 0.01  (-0.07, 0.08) |
| **Country-level variables** |  |  |  |  |
| *NRA tradable agriculture (10%)* | **0.04****  **(0.01, 0.06)** | *0.04*  *(-0.01, 0.08)* | *0.04*  *(-0.01, 0.08)* | **0.09*****  **(0.05, 0.14)** |
| *Share of tradable agriculture (10%)* |  | -0.00  (-0.08, 0.08) | -0.00  (-0.08, 0.07) | -0.06  (-0.13, 0.01) |
| *Log value of production of agriculture* |  | 0.08  (-0.10, 0.26) | 0.09  (-0.08, 0.27) | -0.01  (-0.19, 0.17) |
| *NRA non-tradable agriculture (10%)* |  | -0.03  (-0.16, 0.10) | -0.02  (-0.15, 0.10) | -0.03  (-0.18, 0.11) |
| *Log official development assistance & aid* |  | -0.04  (-0.11, 0.04) | -0.04  (-0.11, 0.04) | 0.00  (-0.07, 0.07) |
| *Governance (democratization)* |  | 0.01  (-0.02, 0.05) | 0.02  (-0.02, 0.05) | **0.05****  **(0.02, 0.08)** |
| ***Interactions*** |  |  |  |  |
| *Parental occupation*NRA tradable agriculture (10%)* |  |  |  |  |
| Non-agricultural | Ref |  |  |  |
| At least one parent self-employed in agriculture |  |  | -0.01  (-0.02, 0.01) | 0.01  (-0.02, 0.03) |
| At least one wage-earning parent |  |  | 0.02  (-0.01, 0.05) | *0.03*  *(-0.00, 0.05)* |
| Parents unemployed |  |  | 0.01  (-0.01, 0.02) | 0.03  (-0.00, 0.06) |
| *Share tradable agriculture (10%)*NRA tradable agriculture (10%)* |  |  |  | **-0.04*****  **(-0.06, -0.02)** |
| *Parental occupation*Share tradable agriculture (10%)* |  |  |  |  |
| Non-agricultural | Ref |  |  |  |
| At least one parent self-employed in agriculture |  |  |  | 0.02  (-0.00, 0.04) |
| At least one wage-earning parent |  |  |  | -0.03  (-0.07, 0.01) |
| Parents unemployed |  |  |  | 0.00  (-0.02, 0.02) |
| *Parental occupation*Share tradable agriculture (10%)*NRA tradable agriculture (10%)* |  |  |  |  |
| Non-agricultural | Ref |  |  |  |
| At least one parent self-employed in agriculture |  |  |  | -0.00  (-0.01, 0.01) |
| At least one wage-earning parent |  |  |  | -0.01  (-0.03, 0.01) |
| Parents unemployed |  |  |  | -0.01  (-0.02, 0.00) |

Notes: Share of tradable agriculture is centered at 50%. Standard errors clustered by country. *Estimates in italics represent p-values < 0.10.* *** represents p-values <0.05. ** represents p-values < 0.01. *** represents p-values < 0.001.**
